# Addressing ableism in healthcare: integrating critical disability theory into health services research

**DOI:** 10.1186/s12939-025-02721-x

**Published:** 2025-12-09

**Authors:** Ellie Gooderham, Julia Smith, Ruth Lavergne, Rita K. McCracken, Lindsay Hedden

**Affiliations:** 1 Faculty of Health Sciences, Simon Fraser University, 8888 University Drive, Burnaby, BC V5A 1S6 Canada; 2 Department of Family Medicine, Dalhousie University, 402-1465 Brenton Street, Halifax, NS B3J 3T4 Canada; 3 School of Medicine, Simon Fraser University, 13450-102 Avenue, Surrey, BC V3T 0A3 Canada

**Keywords:** Healthcare access framework, Critical disability theory, Health services, Lived/living experience, Intersectionality, Structural violence, Ableism

## Abstract

There are limited frameworks for health services researchers to draw on that engage with critical disability theory while considering health services needs of disabled people, despite the quantity of research about disabled people. Research about people with disabilities without inclusion of their perspectives is directly in conflict with the disability rights movement and perpetuates inequity. One mean to include disabled perspectives in research is through disability theory engagement. Through this non-systematic critical review of existing literature, we position critical disability theory in the context of health services research and exemplify why this is a vital consideration when research investigates people with disabilities. We present four tenets of the theory: lived/living experience, intersectionality, structural violence, and ableism, and explore how these can contextualise disabled experiences of healthcare access. We demonstrate how to apply critical disability theory to the Levesque Framework, a commonly used health services access framework. By bridging the gap of critical disability theory inclusion in health services research, we help shift the needle towards improved disability engagement in research and healthcare access equity for people with disabilities.

## Introduction

People with disabilities are estimated to represent 16% of the world’s population and are more likely than non-disabled people to have comorbid conditions [1–4]. The high rates of comorbidity and subsequent healthcare needs result in people with disabilities being significant users of health services and disproportionately impacted by the systems and structures that dictate healthcare access [1–3, 5–9]. While biological contributors of disability are heterogeneous, it is well documented that disabled people collectively experience disproportionately worse health outcomes and a higher mortality rate compared to non-disabled people, and that social determinants of health play a significant role in this [1–3, 5–7, 10]. 

Disabled people experience significant healthcare access barriers due to social inequities compounded by the likelihood of also belonging to other equity-denied groups, based on their demographic and social identity [4, 11, 12]. Access inequities are driven by discrimination built into healthcare systems as well as large-scale social and interpersonal discrimination [2, 4, 7]. These barriers to healthcare access result in disabled people having more unmet health needs compared to non-disabled people, including less engagement with preventative health services [1, 3, 8]. Health equity for disabled people is dependent on healthcare access and this is highlighted in the *Convention on the Rights of Persons with Disabilities* [1, 13–15]. Despite multiple calls to action and extensive activism by disabled people, policy has been slow to address discrimination, one of the root causes of poorer health outcomes [2, 4, 8, 15–18]. 

Evaluating equity in health services access for people with lived/living experience (LLE) of disability is essential to identify and mitigate inequities [1, 13, 14]. Research examining health services access often rely on frameworks that are generally divided into system- or patient-centred models and provide large-scale contextualisation to examine how people interact with healthcare systems. While system-centred models are still frequently used for evaluating healthcare access, patient-centred approaches are becoming more popular [13]. Levesque et al. [19] brought the patient-centred framework to the forefront, where they utilised past concepts focused on the delivery of healthcare and made significant contributions by conceptualising the demand for healthcare and exploring patients’ “ability” to access health services [13, 19, 20]. Despite this patient-centred approach, the Levesque Framework’s applicability for disabled populations has also been critiqued [13]. Yet, the Levesque Framework is widely used; their article has been cited over 1000 times since publication [20]. 

While health services research has documented the inequities faced by people with disabilities, it has made more limited progress in explicit and critical evaluations of the causes of these inequities or identifying means to overcome them. Thus far health research has primarily viewed disability through the medical perspective, ignoring social and political experiences [18, 21]. The effect of this framing is an overt focus on impairments and less consideration of structural determinants, such as discrimination or access to quality care. While non-disabled researchers can try to understand disabled experiences of healthcare, and the Levesque Framework can help organise elements of access, there is an intangible knowledge that can only be gained through having LLE of disability. Critical disability theory/studies (CDT) is a post-modern and transdisciplinary way to conceptualise disability, originating from people with LLE of disability [22–24]. Through this critical lens, the disabled experience of engaging with medical, social, and political systems can be better understood [23–25]. In this paper, **we advocate for greater integration of CDT in health services research to both advance disability-focused research and help reduce barriers to care for disabled people.**

A 2016 scoping review by Berghs et al. [9] found that public health research has done little to produce evidence-based knowledge that engages with disability theory. [9] The lack of disability theory engagement is seen across the disciplines of health research, such as in medical humanities, rehabilitation and social work, and medical sociology, despite calls for change [26–32]. Berghs et al. [9] further identified the benefits of using CDT in public health to address normative health views which included the overall critical nature of the theory, consideration of power dynamics, the fluid nature of embodiment, intersectionality, and ableism. [9] CDT provides avenues to directly critique oppressive, harmful, and normative health perspectives but there are limited examples of engagement by health services researchers [9, 14, 27, 33–37]. **This paper contributes to filling this gap by introducing CDT as it relates to health services research**,** exploring key concepts that can link the two bodies of scholarship**,** and illustrating one approach to this integration through adaptation of the Levesque Framework on access to healthcare.**

### Positionality and reflexivity

Inferences made in this paper are shaped by the positionality of the researchers. The lead author, EG, a settler White cis woman in Canada, has insider knowledge with living experience of disability, and is a health sciences researcher with a background in social sciences. Based on her informed perspective, both identity-first language (disabled people) and person-first language (people with disabilities) are used to discuss disability in this paper. The decision for interchangeable language was made based on how using identity-first language can be a tool to discuss the power and politics of disability, whereas person-first language maintains the individual as the primary focus which can be more inclusive of intersectional experiences [24, 38, 39]. The remaining research team members are non-disabled, settler White cis women with academic and/or clinical health knowledge. To manage bias in this synthesis, authors practiced reflexivity by openly discussing how their experiences may influence their interpretations of the data [40–43]. 

## CDT in health services research

### Overview of medical and social models of disability theory

Despite a movement of empowerment within disability communities, disability largely continues to be viewed through a medical lens, focused on impairment/health deficits, which is closely tied to the idea of abnormality/normativity, and is enmeshed within medicine/health systems [9, 30, 38, 39, 44, 45]. Oppression is a universal experience for disabled people, historically driven by “a dependency born of powerlessness, poverty, degradation, and institutionalization” [46]. This oppression and discrimination is present throughout society [46, 47]. This oppression has been deeply ingrained within the healthcare community for decades, as evidenced in the Alma-Ata Declaration in 1978 which affirmed health as the “state of complete physical, mental and social wellbeing, and not merely the absence of disease or infirmity” [48]. This is inherently discriminatory because impairment or the presence of a disability is not synonymous with incapacity or poor quality of life [45]. Later social theoretical developments are a response to this persistent, medicalised, and normative perspective [9, 21]. 

Disabled people around the world have long advocated for their rights and these efforts were bringing about notable political and social awareness by the 1990s. The disability rights movement coincided with scholars developing a body of literature on disability based on emancipatory and participatory research that emphasised LLE, resistance to oppression, and empowerment. The success of the disability rights movement’s efforts for greater awareness is exemplified by the UN’s Decade of Disabled Persons (1983–1992) [26, 46, 49, 50]. During this time, disability scholarship began to explore the social identity of disabled people [26, 38, 51]. The social model was largely conceptualised by Oliver and served as a rally point for disabled scholars to share their perspectives leading to the proliferation of the social model [26, 51, 52]. As the social model grew, this challenged the medical model by shifting away from the emphasis on health deficits and refocused the issue on how discrimination and prejudice are the cause of disability. Inherent to the social model was identifying and naming how barriers at structural and social levels were the drivers of disability. For disabled people’s health, there was less focus on curing impairments or interventions, and emphasis was made on the prevention of poor health through primary care and social structural change [23, 44]. 

The largest contribution of the social model has been the focus on the social reality of disability. While this is an important development, ignoring the medical aspect of impairment is also not an adequate solution. By trying to move beyond medicalisation of disability, the social model discounts the medical reality of disability, for example the impact that access to health services has for disabled people [26, 44, 53]. While there has been some shift in nomenclature and blending of social and medical models, this blending is still insufficient to fully conceptualise what disability is. This limitation is seen in the biopsychosocial model or the *International Classification of Functioning*,* Disability*,* and Health*, which is the most accepted conceptualisation [9, 25, 35, 54, 55]. It fails to examine structural barriers while also considering disability holistically as a medical, social, and political reality [3, 26, 35, 56, 57]. This is illustrative of the work that still needs to be done to improve the healthcare system’s relationship with disability. There is a need to further conceptualise how the political, systemic oppression experienced by disabled people is shaped by contextual factors, such as widespread discrimination [23, 35]. Ignoring the depth of experiences of disabled people, how disability is a combined medical, social, and political reality, and how these all contribute to barriers in healthcare access, perpetuates an oppressive and reductive view, that does not provide policymakers with meaningful information on which to base healthcare decisions [44, 49]. 

### Critical disability theory

The concept of CDT began to appear in the 2000s and was more representative of a decolonialist lens through the emphasis on global disability experiences [23–25]. This progression in theory originates from people with LLE who provide context rather than a ‘top down’ approach stemming from academia, seeking to end the distinction between LLE and academic expert knowledge [23, 24]. CDT is directly critical of structural systems that reinforce the medical perspective of needing to cure disabled people and of normative views that suggest disabled people cannot fully participate in society [24, 25]. However as the LLE of disability is unique, CDT does not consider accessing health services for impairment prevention, cure, or rehabilitation as inherently negative, but that it can be a morally complex, personal choice [24, 58, 59]. CDT aims to end the co-opting of disabled experiences in systems and institutions to deconstruct the colonialist and medical lenses that have imbued disability with meaning [23, 25]. 

Emerging from the earlier social model, much of CDT scholarship continues to identify social barriers and extends this to theorising why these barriers occur, engaging with concepts including embodiment, agency, and identity [22, 23]. CDT does not discard earlier valuable contributions in disability theories but moves beyond the binary approach of the medical and social models to evaluate structural and systemic oppression [22, 24, 25, 58]. However like previous theory models, CDT is not a perfect means of conceptualising disability, including, reflective of its roots in broader critical theories, lacking a cohesive definition [9, 53, 58]. CDT is well-suited for advocating for empowerment and change, and flaws in the theory can be addressed and improved as CDT is not stagnant. This is reflected in CDT’s transdisciplinary nature, where it incorporates other methods, theories, and frameworks, including those from other marginalised groups [22–25]. CDT uses both lived expertise and academic knowledge perspectives in its critique of oppression, power, and politics. CDT moves to deconstruct normative and medicalised views that have resulted in an ableist society and structural systems that harm disabled people [22–25, 60]. 

To address CDT’s limitation of lacking a cohesive definition and to support its adoption into health services research, we have sought to define CDT in this paper in a brief yet wide scoping manner, that allows for evaluation of the medical, social, and political experiences of disability. We conceptualise CDT as **a tool to critically examine how the identity of being disabled impacts people.** Using a CDT lens in health services research allows us to question the root causes of healthcare inequities for disabled people. **Here we explore four concepts that cut across CDT and health services research**,** providing a basis to bring the two fields of scholarship together: inclusion of LLE in research**,** intersectionality**,** structural violence**,** and ableism.**

#### Lived/Living experience

Ignoring the LLE of disabled people who regularly engage in health services limits the transformative potential of health services research [30]. An insufficient representation of disabled people in research as knowledge holders or through patient engagement initiatives results in a lack of evidence-based decision making [61–63]. This is likely further compounded by exclusion of people with LLE of disability in academic, research, or clinical medicine roles [39, 64]. The lack of integration of disabled perspectives into health services research is likely one reason why health equity for disabled people has not improved [64]. 

**Fig. 1:**
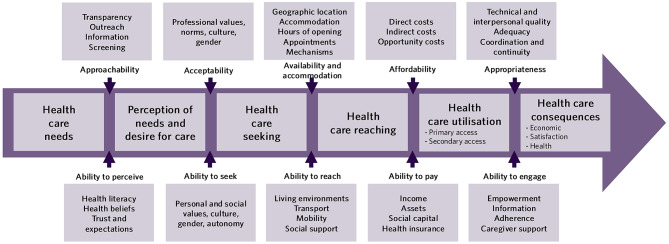
Conceptual framework by Levesque et al. [19] adapted under the Creative Commons CC BY licence

There is a well-documented gap in the integration of disability theory and perspectives from people with disabilities in health services research [9, 21, 62, 65–68]. This is despite the aforementioned integral role that LLE has in CDT and the large representation disabled people have as health services users [1, 24, 46]. Health services research that embeds the perspectives of people with LLE is often described as *patient-engaged* or *patient-oriented research* [69]. This idea of inclusion of LLE can be further extended into *community-based participatory research* and *critical patient-orientated research* where engagement more is collaborative and equity-based [70, 71]. Meaningful engagement is essential to develop patient-centred care models, informed health systems, and improved healthcare access; however, poorly-executed engagement can be superficial, tokenistic, or harmful [61, 63, 72–74]. Snow [63] highlights how health services research needs to consider how engagement occurs in research and the power dynamics that can moderate participation. Figure 1 in their article [63] serves as an excellent example of the types of power dynamics health services researchers need to consider in order to design research that meaningfully includes LLE. Shimmin et al. [75] includes a series of questions surrounding LLE that researchers should consider while conducting health services research. In addition to these resources, we encourage researchers to consider how describing people with LLE as *patients* is not aligned with health equity or disability language principles [76–78]. We suggest that engagement with people with LLE of disability is described to recognise the depth and dimensions a person’s LLE carries [74, 78]. CDT emphasises how integral LLE is to the theory, enshrined in the disability rights movement’s expression of *nothing about us without us* [24, 46]. Therefore within health services research, it is vital to uphold these principles by conducting genuine engagement with people’s LLE of disability.

#### Intersectionality

Disability oppression is often intersectional with other marginalised identities causing greater inequities [3, 6, 23, 25, 46]. The concept of intersectionality developed out of Black feminist activism, and is attributed to Crenshaw who critiqued feminist and racial justice theories that did not recognise the intersecting forces of oppression experienced by Black women in the United States. Intersectional scholarship emphasises understanding the interplay of identities such as race, gender, sexual orientation, socioeconomic status, and disability at the individual-level to reveal interlocking structural inequities. Key principles include recognising relationality, privileging marginalised experiences, exposing intersecting oppressions, contextualising research within contexts, and informing more equitable responses [79–81]. 

Health inequities increase when a person experiences intersectional forms of marginalisation, due to increased oppression and thus barriers to healthcare [6]. Critical theories affirm that to gain equity, structural inequities must be addressed, and the social identity linked to these inequities cannot be ignored. CDT often incorporates intersectionality, while particularly focusing on the diverse LLE of disabled people [24, 25]. Intersectionality within the disability community is complex because disability is heterogenous (i.e. people can be disabled from a variety of causes) and disabled people come from diverse backgrounds (e.g. different gender identities, race, socioeconomic classes, and ethnicities) [3, 6, 23, 25]. 

This means for health scholars, a broad stroke application of addressing barriers in healthcare for disabled people does not work, and interventions to improve health equity must take intersectionality into account. This builds off of LLE, where first the diversity of a person’s experiences must be recognised in order to then understand how the intersection of marginalised identities compounds power dynamics that influence health services research [6, 63, 75]. As Macgregor et al. [6] highlight, the onus is on health services researchers to educate themselves on intersectionality and how using an intersectional approach improves researchers’ understanding of healthcare experiences.

#### Structural violence

Disabled people experience inequity on a structural level due to economic, social, political, legal, and cultural barriers [24, 46]. Health systems are one of these structures and are not established with disabled people in mind [11, 82]. The concept of structural violence – introduced by Galtung and further conceptualised in terms of health inequities by Farmer – provides a conceptual framework for understandings the root causes of inequities [47, 83, 84]. It is similar to the concept of social determinants of health, in recognising that social systems can lead to health inequities and poor health, but differs in that structural violence is explicitly active and political, highlighting the structures as the source of inequities and how they result in violence in terms of physical and mental suffering [47, 84]. Farmer et al. [85] describe structural violence as “social arrangements that put individuals and populations in harm’s way”. [85] The conceptualisation of structural violence has been further expanded to show how the structure of society, be that political, economic, or social, are hegemonic and stratify access, leading to health inequities which are harmful and thus violent [47, 85]. Unlike the concept of social determinants of health, which has become pervasive in public health scholarship, structural violence has been applied more frequently in medical anthropology [84]. There is a movement to further incorporate structural violence into health services research with disabled populations [86–90]. Yet, the full intersection of CDT, health services, and structural violence is limited despite how CDT directly critiques structural systems, examines society, and advocates for the dismantling of inequity [22, 25, 91]. 

In health services research it is imperative to consider how these systems are structured and operate, and how this organisation can increase barriers for people with disability. This can be done for example, through critical patient orientated research since it is situated within health systems, allowing for better evaluations of structural harms [70]. In sum, health services researchers should learn about the structural violence health systems perpetuate against disabled people through engagement with people with LLE as knowledge experts.

#### Ableism

Ableism is the oppression of disabled people built upon the concept of normativity [39, 91–93]. Fine describes ableism as “structural, social, political, institutional, interpersonal, and intrapsychic violence; a dynamic so naturalized that it is hard to see and difficult to name, saturating how we organize work, public policies and institutions, schools, identities, criminal punishment, welfare, personal relationships, and everyday life” [91]. The violence described is also an expression of structural violence, yet is goes beyond this, as ableism also considers the deeply rooted social acceptance of oppression of disabled people [91]. Ableist perspectives promote that disabled people cannot be independent, are vulnerable, and must be rehabilitated, cured, or eliminated [23, 45, 92]. Disabled people are discriminated against by non-disabled people because they represent fears of losing ability, are disruptive to normative views, and are considered burdens [22, 94]. Ableism can be internalised, due to social norms or experiences in society [93]. Furthermore, CDT highlights the harms of ableist attitudes of pity and charity [24]. 

Investigating ableism in health services research to combat its deep roots in health systems is vital and the ableist nature of healthcare is seen through a multitude of examples. Applying a CDT lens through critiquing power and oppression is a means to address this. In healthcare, ableism can occur due to healthcare providers’ biases that play out in interpersonal patient-provider interactions (e.g. blaming patients, dismissing disabled people’s LLE, and infantilising them), or as microinequities (e.g. not providing accessible healthcare spaces) [39, 45, 95–99]. Ableism in healthcare is also structural, as it is naturalised into health policies, law, and medical school curricula [9, 39, 45, 93, 99]. Structural ableism has been seen during emergencies such as natural disasters (e.g. Hurricane Katrina) or health emergencies (e.g. COVID-19) where disabled people were deprioritised [59, 100, 101]. The most extreme form of ableism in healthcare is eugenics, which can take on the role of preventing or eliminating disability, for example, through coercive medically assisted death [39, 59]. Today, eugenic practices are mostly decided by family members or social services and preventing disability is often through abortion, or non-consensual sterilisation [24, 39, 44, 59, 93, 102, 103]. Health services researchers must take into account how ableism and normativity are accepted as the status quo and through applying CDT to their work, evaluate this.

## Applying CDT to the Levesque framework

Levesque et al. [19] understood health services access as the interaction between individuals and populations with health system structures across the lifecycle of access, from identification of need to the consequences of receiving healthcare. This process is illustrated in Levesque and colleagues’ [19] framework (Fig. 1). By focusing on the patient’s trajectory through a series of stages (needing, seeking, reaching, using, and results of healthcare) that are moderated by ‘dimensions’ impacting this pathway, researchers can evaluate where along this pathway their studied health system is failing or serving the needs of their population. The Levesque Framework visualised how dimensions act as barriers or facilitators to access along the pathway. These dimensions are introduced on one side by the health system including healthcare providers and on the other side, the individual patients whose LLE moderate their ability to follow the pathway. They explained that in the delivery of health services, barriers are introduced by healthcare providers, processes of care, the quality and availability of services, and structural systems such as government policies. [19]

Levesque et al. [19] suggested that by evaluating variation in health services utilisation, researchers could also assess inequities in healthcare access. Their critical approach emphasised the patient experience, equity, and social determinants of health. Further, they highlighted that theoretical healthcare access does not equate to lived experiences. In future direction suggested by the authors, they provided avenues for updating their framework in consideration of different social groups. [19] Despite the strengths of the Levesque Framework, it does not fit the needs of all healthcare research, and researchers who rely on it find both benefits and shortcomings within the framework. Critiques or adaptations to the Levesque Framework can be found in literature for various equity-denied groups. This is not surprising as no framework can capture all aspects of all health systems worldwide, as each health system is a product of distinct political and social environments [13, 20, 104–106]. 

The specific limitations of the framework have also been highlighted in scholarship that situates the LLE of disability when accessing health services. Nguyen et al. [13] questioned the utility of the Levesque Framework from the position of disability studies and social science scholarship, highlighting how health system access frameworks (including the Levesque Framework) do not sufficiently engage with the complexity and LLE of disability for evaluating healthcare access. The authors suggested that, despite the patient-centred approach of the Levesque Framework, it lacks the ability to capture the nuances of disability, especially in low or middle income countries. [13] Similarly, Schwarz et al. [67] noted that the Levesque Framework does not fully capture accessibility barriers caused by a fragmented healthcare system in a high income country. They emphasised how fragmented health services created challenges for individuals with chronic illnesses who likely experience more than one illness and further highlighted the current gap in research that connects structural barriers to the lived experiences of disabled people. [67] Sakellariou et al. [33] engaged with CDT and the Levesque Framework together. The authors were able to use CDT to expound on how barriers were examples of ableism and discrimination. [33] Collectively, these researchers all demonstrate the ways in which the Levesque Framework can aid our understanding of health service access for disabled people, but also that the framework fails to capture essential aspects of disability.

**Fig. 2:**
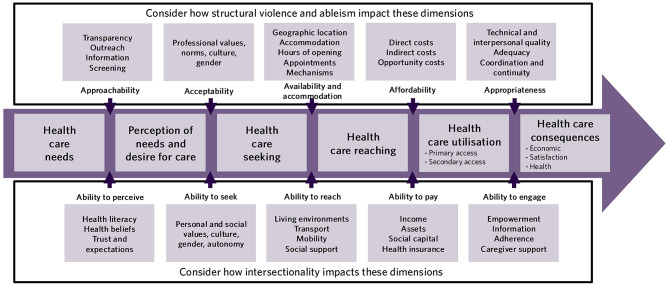
A critical disability theory informed Levesque framework

The limitations of the Levesque Framework, in terms of reflecting and responding to health inequities experienced by disabled people, can be partly addressed by incorporating elements of CDT – particularly the concepts of intersectionality, structural violence, and ableism. Paramount to this is how a person’s LLE (including of experiences of intersectionality, structural violence, and ableism) impacts the entire process of healthcare access (see Fig. 2). In order to leverage the strengths of the Levesque Framework when conducting health services research with disabled population, the disabled experience must be at the forefront of consideration and research must integrate the critical role of LLE of disability [61–63]. We suggest health services researchers consider how structural systems or discriminatory perspectives can impact how healthcare is delivered [39, 45]. The legacy of medicalisation of disability has lasting influence that continues to shape health systems [9, 38, 44, 47]. Structural violence allows researchers to evaluate how the structuring of health systems influence access to and delivery of health services [85, 87]. Viewing the Levesque Framework and its dimensions with this knowledge, researchers can critique the ways that the political, social, and legal foundations of healthcare systems (i.e. the delivery dimensions in the Levesque Framework) result in a disproportionally difficult experience of accessing care for disabled people. For example, it is well documented that disabled people are less often offered appropriate health services (Approachability dimension), sometimes due to ableist perspectives of healthcare providers (Acceptability dimension) [11, 45, 99]. It is further known that health services can operate in built environments that are not accessible (Availability and accommodation dimension) [11, 99]. Researchers must also consider how disabled people’s identity/ies and experiences can influence their health service use. For people with disabilities accessing care, researchers can incorporate an intersectional lens in their health services evaluation (i.e. across the five Ability dimensions) [6, 63, 75]. For disabled people, historic and lasting oppression has greatly influenced their trust (Ability to perceive dimension) in healthcare systems and healthcare professionals [45, 95, 107]. Furthermore, infantilisation and other biased views try to erode disabled people’s autonomy (Ability to seek dimension) and empowerment (Ability to engage dimension) [39, 95, 107]. These experiences can intersect by the rate in which disabled people are further marginalised by poverty and experience greater healthcare costs (Ability to pay dimension) [107]. 

At first glance the Levesque Framework seems to capture the essential elements of the determinants of healthcare access; however, for the disability community who represent 16% of the world’s population, the framework does not capture the complexity of accessing services. In order to improve health services for disabled people, researchers need to better capture the root causes of access inequities [7, 18, 108]. Discrimination and oppression directly influence health-seeking behaviours and how health services are delivered to disabled people. The examples we provide within each Levesque dimension represent both nuanced and more apparent experiences of disability. To actualise this into research practice, we recommend a CDT-informed approach with explicit engagement with LLE regardless of which access framework other tools health services researchers are using.

### Moving forward in disability-oriented health services research

In questioning the suitability of the Levesque Framework for evaluating health services access for disabled people in its current form, we caution that as researchers, we must understand the limitations of our tools. Imperfections in frameworks can lead to less impactful findings and lessens our ability to take appropriate action [109]. As Szymczak et al. [106] suggest, the Levesque Framework can be taken from a place that identifies healthcare access inequity, to a critical tool to address it. [106] The trickle-down impact of evolving the Levesque Framework is that research that engages with it will be more aligned to the needs of health equity-denied groups, such as disabled people. Despite its limitations, since the Levesque Framework is regarded as patient-centred and comprehensive and disabled people are significant users of health services, there is much potential in adapting this framework to better consider the access needs of people with disabilities [1, 8, 13, 19, 20]. 

We do not suggest that modifications to the Levesque Framework should be ‘one size fits all’. Drawing on CDT to better understand the LLE of disabled people can strengthen the ability of the framework to reflect the needs of people with disabilities in the specific research environment under investigation. Further, engaging with disability-oriented health systems frameworks can supplement researcher’s knowledge of barriers in healthcare access for disabled people. This is a growing topic, with two notable examples published in 2024 [7, 18]. 

Understanding the ways the Levesque Framework is not sufficient in conceptualising healthcare access for people with disabilities is important, as the research using this framework informs policy that shapes healthcare systems. However, the Levesque Framework is just one of many examples of health services research that ignores the LLE of the population under investigation. We strongly advocate for health services research to actively engage with CDT and people with LLE when conducting research about people with disabilities. CDT is an explicitly fluid approach that has the potential to inform equity focused health services research more broadly. Here we have presented one example of applying key CDT concepts of LLE, intersectionality, structural violence, and ableism to a specific framework – this is just a starting place. There is much potential to integrate CDT more broadly in health services research and consider how it might inform other common frameworks, particularly when research engages with topics related to disability. Greater integration of CDT, particularly its insistence on prioritising LLE, would align with the disability rights movement principle of *nothing about us without us* [46]. Seeking better engagement with disabled people during research will further draw on CDT core tenets of embodiment, empowerment, and identity. Naming and engaging with CDT during research is a powerful way of directly critiquing health services and by identifying marginalising aspects of these services, researchers can support progress in disability health equity. Explicit naming of CDT as an applied theory can occur in research in a variety of spaces, such as the introduction, background, methodology, or positionality statements. In this action of emancipation, researchers are disseminating knowledge about the theory to others who are unaware of it – as we have attempted to do with this article – and this provides avenues for further engagement.

## Conclusion

The exclusion of CDT from health services scholarship results in less impactful research that is centred on the medical model of disability. As disability research from the social perspective has demonstrated, changes to improve health services cannot solely come from a medicalised perspective. CDT can be used to examine why disabled people experience health inequities, through evaluating the structure of health systems and services and advocating for evidence-based decision making. Our perspective is exemplified in our review of the Levesque Framework through the lens of CDT. We identified that the Levesque Framework does not fully capture the healthcare access needs of disabled people and point how CDT can be used to evolve the framework in ways that overcome this limitation. We encourage other health services researchers to engage with CDT to design, facilitate, and report studies that are centred around disabled people. The effort to understand the LLE of disabled people is especially important when research is being conducted on disabled populations by non-disabled scholars. We suggest that scholars consider how structural violence and ableism are barriers in healthcare access for people with disability and that intersectionality highlights further healthcare inequities. We argue that the neglect of CDT in health services research is a detriment to the discipline and its inclusion could lead to improved healthcare policies.

## Data Availability

No datasets were generated or analysed during the current study.

## Abbreviations

LLE: Lived/living experience
CDT: Critical disability theory/studies
